# Long COVID Cardiopulmonary Symptoms and Health Resort Treatment: A Retrospective Study

**DOI:** 10.3390/jcm13185563

**Published:** 2024-09-19

**Authors:** Grzegorz Onik, Katarzyna Knapik, Karolina Sieroń

**Affiliations:** School of Health Sciences in Katowice, Medical University of Silesia in Katowice, Department of Physical Medicine, Chair of Physiotherapy, 40-055 Katowice, Poland

**Keywords:** long COVID, mMRC, dyspnoea, symptom severity, balneotherapy, health resort treatment

## Abstract

**Background/Objectives**: Long COVID covers many cardio-pulmonary symptoms, worsening individuals’ health status. Health resort treatment applies balneological factors, physical medicine modalities, climate actions, and exercises that may be beneficial for COVID-19 survivors. This study aimed to assess the severity of the cardiopulmonary symptoms in people qualified for health resort treatment and its efficacy in this group of patients. **Methods**: Medical records of 239 people attending health resort treatment were analysed. A total of 122 people (71 women and 51 men) with a mean age of 64.35 years ± 8.66 years were enrolled in the analysis. The cardiopulmonary symptoms of long COVID were assessed twice: before and after health resort treatment. **Results**: Persisting COVID-19 symptoms do not differentiate between women and men. Health resort treatment reduces symptoms severity in both sexes. Age does not mediate the efficacy of health resort treatment. **Conclusions**: The persistent symptoms of COVID-19 are of low intensity in people qualified for health resort treatment and are independent of gender. Health resort treatment effectively mitigates dyspnoea, tightness of chest, and sputum in long COVID patients, so it should be implemented into the standard treatment course for COVID-19 survivors as a continuation of therapy.

## 1. Introduction

Long COVID refers to symptoms of SARS-CoV-2 infection that persist despite the acute phase being over [1,2]. The World Health Organization defines it as a condition occurring in individuals with a history of probable or confirmed SARS-CoV-2 infection, usually three months from the onset of COVID-19, with symptoms that last for at least two months and cannot be explained by an alternative diagnosis [1,3]. Long COVID is estimated to occur in 10–20% of COVID-19 survivors, with the highest incidence in people aged 36–50 years [4,5]. Among other factors, old age, female sex, and pre-existing medical conditions are listed as predictors of long COVID [3,4,5].

Symptoms of cardiovascular, pulmonary, nervous, and gastrointestinal systems disorders, as well as mental ones, may manifest in people with long COVID [1,4,5,6,7,8]. The most frequently listed cardiopulmonary symptoms of long COVID are dyspnoea, coughing, tachycardia/palpitations, chest pain, chest tightness, and sputum [1,3,4,5,6]. A probable mechanism to explain the persisting symptoms of COVID-19 assumes prolonged inflammation arising from organ damage [5]. Other hypotheses claim that immune system disorders, antibody generations, hypoxia-dependent coagulopathy, and endothelial dysfunction play a role in the pathomechanism of long COVID [8,9,10,11]. Some researchers have also postulated that autonomic dysregulation determines the persistence of COVID-19 symptoms [1,10].

The quality of life is reduced in people with long COVID, as convalescents may manifest different symptoms [12,13]. That is why different treatment strategies are implemented to alleviate the symptoms, including supportive pharmacotherapy [1,4,5,7,8,10,11], rehabilitation with general and respiratory exercises [6,7,9,10,14,15], diet supplementation [1,10], and hyperbaric oxygen therapy [16]. The European SPA Association suggests that balneotherapy may improve health status and mitigate the symptoms in people with long COVID [17]. As they manifest symptoms similar to those observed in cardiopulmonary disorders in which balneotherapy and climatotherapy were proven to be beneficial [1,3,4,18,19,20,21], it might be assumed that this method of treatment may effectively act in people dealing with persisting COVID-19 symptoms. Nevertheless, few reports are devoted to the health-resort treatment in long COVID, and most of them are theoretical considerations [17,22,23,24]. The only paper assessing the efficacy of the health resort treatment in people with persisting COVID-19 symptoms was published by Gvozdjáková et al. [25], but the sample size was small. Complex treatment in health resorts includes a wide range of procedures, such as massage, hydrotherapy, mineral waters, physical medicine modalities, mud baths, and different exercises that may be beneficial for patients with long COVID. Moreover, the effects of health resort treatment are augmented with climate action, as medical resorts are located in areas meeting the criteria for a healing climate [17,22,23,24]. That is why our retrospective study fills the knowledge gap by assessing the efficacy of health resort treatment in people with long COVID.

This study had two aims. Firstly, it aimed to assess the persistence of COVID-19 symptoms in people qualified for health resort treatment. Secondly, it sought to assess the effectiveness of the health resort treatment in people with long COVID.

## 2. Materials and Methods

### 2.1. Methods

Data for this retrospective study were collected between March and May 2024 in the Rehabilitation Hospital and Sanatorium “Gwarek” in Goczałkowice-Zdrój, Poland. Medical records of 239 people who had recovered from COVID-19 and underwent health resort treatment in 2021 because of long COVID were analysed. All patients that qualified for health resort treatment had to have recovered from SARS-CoV-2 infection within twelve months before admission due to the Polish legal regulations of the post-COVID rehabilitation program.

The severity of the long COVID symptoms was assessed twice: during medical examination at admission and discharge. Each patient underwent a check-up after seven days of treatment to confirm a positive reaction to the applied treatment. Final examination confirming improvement and completion of the assumed treatment program were the criteria for discharge. In-patients assessed their persisting COVID-19 symptoms using a 0–10 scale, where zero meant “no symptoms”, and ten meant “the most severe symptoms imaginable”. The following symptoms were assessed: dyspnoea at rest, exercise-induced dyspnoea, cough intensity, tightness of the chest, chest pain, sputum, palpitations, and fast heart rate. The dyspnoea was also assessed using the modified Medical Research Council (mMRC) scale. This five-point scale enables qualitative assessment of dyspnoea-mediated disability in daily routines. Zero means “dyspnea during intensive effort”, and ten means “inability to wear and leave home because of dyspnea” [26,27,28,29]. The Bioethical Committee of the Medical University of Silesia in Katowice stated that this project does not require its approval (decision number: BNW/NWN/0052/KB/238/23).

Between the measurements, all participants attended complex health resort treatment, including balneotherapy, exercises, physical medicine modalities, and health education. The treatment program was designed individually based on the clinical examination performed at admission. In all participants, respiratory exercises were implemented. They included relaxation exercises (stretching of chest muscles), extended exhalation, diaphragmatic breathing, and lower ribcage activation. One hundred twenty-one people (99.18% of participants) performed the general development exercises. The exercises aimed to improve dynamics, endurance, balance, coordination, and muscle strength. A proportion of 65.57% of participants attended pneumatic massage, whereas classical massage was prescribed to 5.73% of patients. Only one person had lymphatic drainage performed (0.82% of participants). In 59.84% of participants, whirlpool baths were prescribed, while pearl baths (a mineral water bath with compressed air micro-massage) were also included. Mud therapy was applied in 13.94% of patients. During the pre-treatment examination, patients listed other medical problems, primarily musculoskeletal, as legal acts recommended rehabilitation of coexisting conditions in long COVID patients undergoing health resort treatment. That is why physical medicine modalities were widely administered. Low-level laser therapy was the most commonly applied modality (59.02% of participants). A proportion of 40.98% of patients were exposed to infrared light. Light therapy using infrared was performed in 40.98% of patients. Local cryotherapy was applied in 50.82% of patients. Electromagnetic fields were administered in 37.7% of patients, while electrotherapy (mainly transcutaneous electrical nerve stimulation) was applied in 34.43% of patients. Ultrasounds were applied less frequently in the study group (4.92% of people). All participants received health education emphasizing bad habit breaking, advice on a healthy diet, physical activity recommendations, and learning the correct technique of using inhalers, if needed.

### 2.2. Characteristics of Participants

A total of 89 people were disqualified from further analysis because they met the exclusion criteria. The following exclusion criteria were set: age above 80 years, neuropsychiatric disorders (multiple sclerosis, post-stroke syndrome, Parkinson’s disease, epilepsy, depression), cardiovascular system diseases (coronary artery disease, heart failure, myocardial infarction in history, percutaneous coronary interventions and/or coronary artery bypass grafting in history, endarterectomy in history, pacemaker, atrioventricular and/or bundle of His blocks, atrial fibrillation, peripheral artery disease), respiratory system disorders (chronic obstructive pulmonary disease, emphysema, pneumoconiosis, asthma), rheumatic diseases (rheumatoid arthritis, ankylosing spondylitis), cancer, lower limb amputations, blindness, and Lyme disease. The remaining group of 150 people was included in the subsequent analysis stage. An additional 28 participants were excluded from the analysis because of incomplete data to mitigate the risk of sampling bias.

### 2.3. Statistical Analysis

Statistical analysis was performed using STATISICA 13 PL software. Qualitative variables are presented as percentages. Quantitative variables are presented as means with standard deviations. The effect size was calculated using G Power 3.1.94 software. With the assumption of the sample size 122, α = 0.05 and statistical power (1-β error probability) = 0.95, the effect size was established as ƒ = 0.306. Data normalcy distribution was checked with the Shapiro–Wilk test. Intragroup comparisons were performed using the Wilcoxon signed-rank test. The homogeneity of variance was checked with Leven’s test. Kruskal–Wallis one-way analysis of variance by ranks with post-hoc tests was used to perform intergroup comparisons. We calculated the ∆mMRC expressing the difference between the mMRC scale scores reported by individuals before and after health resort treatment. The statistical significance level was set at *p* < 0.05.

## 3. Results

The study group consisted of 122 people (71 women and 51 men) aged 42–79 years (mean age: 64.35 ± 8.66 years). The mean body weight of the participants was 85.73 kg ± 15.22 kg, the mean body height was 1.67 ± 0.09 m, and the mean body mass index was 30.64 ± 5.08 kg/m^2^. The mean systolic blood pressure in the study group was 139.77 ± 13.28 mmHg, and the diastolic one was 80.06 ± 7.52 mmHg, measured on admission day. The mean duration of the health resort treatment was 24.59 ± 6.38 days. Polish legal rules enabled 2–6 weeks of treatment. The most frequently health resort treatment lasted 21 days (54.92% of participants). In 3.28% of participants, it was shorter than 21 days, while in 44.8% of patients, it was longer. Hypertension was the most common comorbidity in the study group. It was noted in 70 participants (57.37% of the study group). In hypertensive people, the mean systolic blood pressure was 141.8 ± 13.12 mmHg, whereas the mean diastolic one was 80.27 ± 8.16 mmHg during the pre-treatment examination. Type 2 diabetes mellitus occurred in 23 people (18.85% of the study group). In diabetic patients, the mean glycaemia at admission was 122.69 ± 18.46 mg/dL. A proportion of 13.11% of people were diagnosed with hypothyroidism and four with gout (3.27% of participants). Osteoarthritis was diagnosed in 22.13% of patients. Participants’ characteristics and the incidence of comorbidities are presented in Table 1.

The study group was divided into three subgroups according to the age range to prevent selection bias, so that the group might be representative of the broader population. Group I consisted of people aged 40–59 years (N = 37; mean age: 53.59 ± 5.36 years). In group II, people with an age range of 60–69 years (N = 42; mean age: 65.00 ± 2.9 years) were included. Participants aged 70–79 years were included in group III (N = 43; mean age: 72.98 ± 2.23 years). Characteristics of the age-dependent groups are presented in Table 2.

During the pre-treatment measurement, participants assessed their dyspnoea with an mMRC scale from 0 to 4 points (mean: 1.37 ± 0.95 points), while after the health resort treatment, the highest score was 3 points. After the health resort treatment, the mMRC scale scores revealed a 62% decrease in the study group (mean: 0.52 ± 0.73 points) (*p* < 0.0001). The subjective assessment of persisting COVID-19 symptoms severity decreased significantly during post-treatment measurement, except for fast heart rate. Sputum and cough intensity decreased by 82% and 81%, respectively. Participants declared the highest drop in chest pain (77% decrease) and chest tightness (73% decrease) among cardiac symptoms. Dyspnoea at rest diminished by about 67%, while exercise-induced dyspnoea reduced by about 63%. Table 3 presents the severity of the long COVID symptoms during pre- and post-treatment measurements.

**Figure 1:**
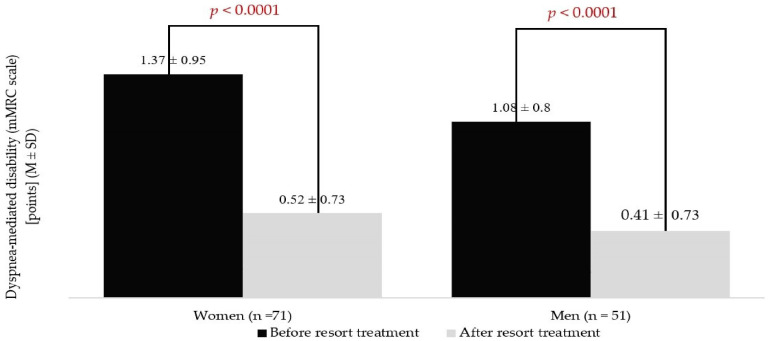
Results of the modified Medical Research Council (mMRC) scale in women and men during pre- and post-treatment measurements.

In women, the mMRC scale scores were statistically higher compared to men during the measurement performed at admission (*p* = 0.007). After the health resort treatment, the mMRC scale scores were about 62% lower in both groups. Women did not differ significantly from men during post-treatment assessment (Figure 1). In women, the mean ∆mMRC (0.99 ± 0.77 points) was higher in comparison to the men (mean: 0.66 ± 0.65 points) (*p* = 0.03).

Before the health resort treatment, no significant differences between sexes in long COVID symptoms severity were noted. The remaining symptom severity was lowered in both genders due to the medical resort treatment, except for fast heart rate. Moreover, palpitations decreased significantly after health resort treatment only in women (68% diminishment). Dyspnoea at rest decreased by about 67.5% in women and about 80% in men. Exercise-induced dyspnoea in both sexes decreased by about 63%. Decreases in cough intensity and sputum were comparable in women and men, with reductions of 81% and 80%, respectively. The tightness of the chest was reduced by about 75% in women, while in men, it was reduced by about 68%. Even though the chest pain intensity did not differ significantly between sexes, in men, the decrease was higher (83%) compared to women (68%). After staying in the sanatorium, fast heart rate was significantly lower in men compared to women (*p* = 0.02) (Table 4).

**Figure 2:**
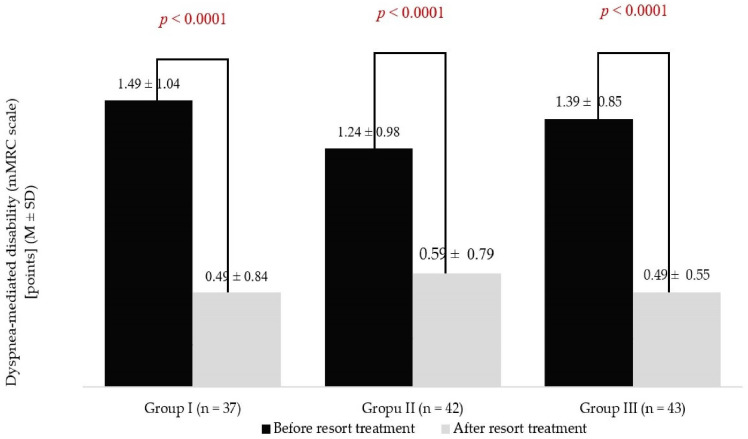
Results of the modified Medical Research Council (mMRC) scale during pre- and post-treatment measurements in the age groups.

Dyspnoea, assessed with the mMRC scale, did not differ significantly between the age-dependent groups during pre- and post-treatment measurements. Nevertheless, in each age group, statistically significant decreases in the mMRC scale scores were noted (Figure 2). The highest mean ∆mMRC was noted in people aged 40–59 years (1.00 ± 0.82 points), while the lowest was in patients aged 60–69 years (0.64 ± 0.69 points). The mean ∆mMRC in the oldest group of patients was 0.91 ± 0.66 points. No significant differences between the age groups were noted (*p* = 0.11).

The comparison of symptom severity between the groups depending on age range did not reveal statistical significance. All age groups noted statistically significant reductions in dyspnoea at rest, exercise-induced dyspnoea, cough intensity, and sputum. Even though in people aged 50–59 years, tightness of the chest was reduced by about 73% after the resort treatment, this result was not statistically significant. The reductions in chest pain, fast heart rate, and palpitations were statistically significant only in the youngest participants, with values of 82%, 88%, and 72.5%, respectively (Table 5).

**Figure 3:**
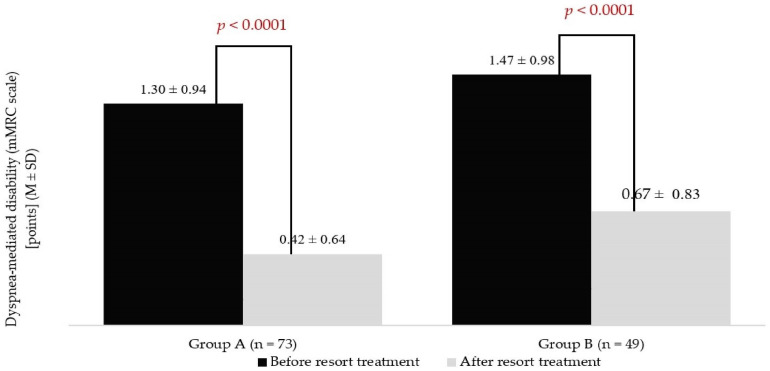
Results of modified Medical Research Council (mMRC) scale during pre- and post-treatment measurements in groups of patients based on treatment strategy.

The study group was divided into two subgroups depending on the application of balneological factors. Group A comprised patients receiving mineral baths or mud therapy integrated into the treatment course. Group B included people undergoing health resort treatment without application of natural healing resources. Dyspnoea-mediated disability assessed with the mMRC scale decreased by about 66% (*p* < 0.0001) in group A, and by about 55% in group B (*p* < 0.0001) (Figure 3). The mMRC scale scores did not differ significantly between the groups at either stages of this study. The calculated mean ∆mMRC in patients treated with natural healing resources was 0.88 ± 0.78 points, while in people included in group B, it was 0.79 ± 0.68 points. The groups did not differ significantly (*p* = 0.59).

Dyspnoea at rest decreased by about 75% in the people from group A and by about 65% in group B. Exertional dyspnoea reduction was higher in group A compared to group B, respectively, 66% and 62%. Similarly, the decrease in cough intensity was higher in group A (84%) compared to group B (78%). On the contrary, tightness of the chest and chest pain decreases were higher in group B (78% and 92%). Group A had a higher reduction in sputum (about 85% reduction). In both groups, heart palpitations remained unchanged after the health resort treatment. Even though in group A, chest pain was reduced by about 58%, it was not statistically significant. In group B, palpitations after the stay were reduced by about 78%; however, this was not statistically significant. The effects of complex treatment based on treatment strategy are presented in Table 6.

## 4. Discussion

A key finding of this study is that health resort treatment is beneficial for people with long COVID cardiopulmonary symptoms. The baseline mMRC scale scores in the study group (1.37 ± 0.95 points) were lower than those presented by Romanet et al. [30]; however, they excluded people with little or no dyspnoea (mMRC ≤ 1 point). In our study, 56.57% of participants declared little or no dyspnoea at admission (mMRC ≤ 1 point). Our results are consistent with Ma et al. [31] and Sperling et al. [32], who reported that most patients report mMRC = 0 one year after SARS-CoV-2 infection. The incidence of server dyspnoea (mMRC ≥ 2 points) in our study was higher in comparison to the report by Sperling et al. [32]. During the pre-treatment measurement, the mMRC results were statistically higher in women than in men. The results are inconsistent with the data provided by Ma et al. [31], who found that men are more likely to have dyspnoea. In women enrolled in the analysis, severe dyspnoea (mMRC ≥ 2 points) was declared in 39 (54.93%) of them. In men, only 14 participants complained of severe dyspnoea (27.45%). This may explain the obtained differences. Nevertheless, reports claim that women are more likely to develop long COVID [3,4,5]. In all participants, a treatment-dependent decrease in dyspnoea-mediated disability assessed with the mMRC scale (about 62% drop) was noted. Furthermore, ∆mMRC was higher in women compared to men. It may indicate greater effectiveness of applied treatment in women. However, males had higher mMRC scale scores during the baseline measurement. Romanet et al. [30] proved that exercise training decreased mMRC scale scores in people with long COVID. Moreover, Rao et al. [33] indicated that supervised hospital-based pulmonary rehabilitation improved mMRC scale scores. In our study, all participants received respiratory exercises, and 99.18% of them attended general development exercises and were exposed to balneological factors and physical medicine modalities. This may explain the improvement in mMRC scale scores.

In the study group, the subjective assessment of symptoms severity did not differ significantly between sexes during the baseline measurement. Ma et al. [31] also found that coughing and chest pain do not differ between women and men. Watase et al. [34] suggest that coughing is more common in women than in men. In our study, 34 women (47.89%) and 20 men (39.22%) assessed their cough intensity ≥ 1 during pre-treatment measurement; thus, in the study group, coughing was also more frequent in the female sex. Our baseline results are also consistent with data presented by Bai et al. [35], who found that rest and exertional dyspnoea do not differentiate between females and males with long COVID. Moreover, they also proved that dyspnoea is more common in women, which is also consistent with our results. In our study, 56 women and 41 men declared exertional dyspnoea (≥1 point in this domain). Lai et al. [6] claim that chest tightness affects 48% of long COVID patients. In the study group, 31.25% of participants declared chest tightness severity ≥ 1 point during pre-treatment measurement. The prevalence of sputum was higher in participants compared to the report by Lai et al. [6], who assessed its incidence at 2–4%. However, our results are similar to those of Yong et al. [7], reporting a 43% prevalence of sputum in long COVID people. Lippi et al. [3] report a high incidence of tachycardia and palpitations in people with persisting SARS-CoV-2 infection symptoms. In our study, 89.34% of participants declared no palpitations during the admission (0 points in this domain). The incidence of symptom is in accordance with Yong et al. [7], who report an 11% prevalence. The frequency of chest pain in people with long COVID is diverse, depending on the report. Carfi et al. [36] estimate that chest pain incidence is 21.7% in people about 60 days after the onset of COVID-19 symptoms, whereas Davis et al. [4] indicate a 53% prevalence in people 7 months after infection. In the one-year observation by Huang et al. [37], the chest pain incidence was 7%. In the study group, 15.53% of participants declared chest pain to be present (≥1 point in this domain). Our results are comparable with those in the literature; however, we did not interview participants during the time interval following the active SARS-CoV-2 infection clearance.

Koc et al. [1], Lippi et al. [3], and Cabrera Martimbianco Martimbianco et al. [38] inform that older age is associated with long COVID symptoms, while Perlis et al. specify that older age per decade above 40 years is associated with their persistence [39]. In the study group, no significant differences between age-dependent groups were noted during pre- and post-treatment measurements. People qualified for the health resort treatment must meet legally regulated, distinct criteria, which might explain why there were no differences between the age groups, as all participants had to present the required level of independence and mobility [40]. Health resort treatment was least effective in people aged 60–69 years because improvement was noted only in four domains: dyspnoea at rest, exercise-induced dyspnoea, cough intensity, and sputum retention. However, the ∆mMRC analysis did not reveal significant differences between the age groups. In long COVID symptoms, severity is associated with coexisting conditions [1,3,5,7]. In the study group, comorbidities were the most frequent in people aged 70–79 years: hypertension (n = 28), type 2 diabetes (n = 11), hypothyroidism (n = 7), osteoarthritis (n = 6), and gout (n = 2). Health resort treatment is proven to be beneficial in hypertension, heart failure, and diabetes mellitus [18,19,20,21,41]. That is why it cannot be assumed that coexisting diseases mediated treatment effectiveness in the people enrolled in the health resort treatment. We could not establish if comorbidities had been present before SARS-CoV-2 infection or were mediated by COVID-19. However, the available reports dedicated to rehabilitation in long COVID also included people with hypertension or type 2 diabetes [30,42,43,44]. That supports our study group composition and legitimizes further analysis.

The applied health resort treatment alleviated the long COVID symptoms, except for fast heart rate. In men, the severity of fast heart rate and palpitations remained unchanged. On the contrary, in women, the intensity of palpitations was significantly lower after the treatment at the health resort. The noted alleviation of symptoms severity might result from the complex action of the health resort treatment. Castelli et al. [45] claim that balneological factors may be effectively applied in endothelium-dependent pathologies, as they act on microcirculation and stimulate adaptive reactions in the autonomic nervous, endocrine, immune, and thermoregulation systems. Considering that one of the hypotheses of long COVID assumes endothelial damage, the action on balneological factors may be curative [8,9,10,11]. Moreover, those factors are also proven to act on the autonomic nervous system, which might be dysregulated in long COVID [1,10,46].

Most of the patients enrolled in the analysis had different types of baths administered during the stay, while mud therapy was applied to 13.94% of participants. Hydrotherapy with hot water improves blood flow due to therapy-mediated vasodilatation [47] and reduces the fibrinogen level [48]. Furthermore, the physical properties of water influence inspiratory muscle activity, facilitate expiration, and reduce the residual volume [18]. Mud therapy and balneotherapy are concerned with bi-directional action. They diminish the concentrations of pro-inflammatory cytokines and increase the level of anti-inflammatory ones [49,50]. The statistical analysis revealed that ∆mMRC did not differ significantly between patients who had balneological factors administered (mineral baths and mud therapy) and those not exposed to them. What is more, considering the number of domains that were improved after the health resort treatment, it seems that the efficacy of applied curation is comparable in patients who were treated with natural healing resources and those who were not. Nevertheless, this study’s aim was not to assess the efficacy of particular methods.

The action of balneological factors was supported with respiratory and general exercises. Pulmonary rehabilitation is proven to be beneficial in people with long COVID [30,33,42,51]. The health resort treatment was conducted in Goczałkowice-Zdrój. The town is located in the upper Vistula Valley in the Oświęcim Valley. Thus, it is a lowland resort that obtained a health resort status in 2016 as it has a healing climate, and the presence of balneological factors has been confirmed [40,52]. Kubincová et al. [53] found that climatotherapy positively impacts ventilation parameters and the 6 min walk test results in chronic obstructive pulmonary disease patients. To the best of our knowledge, there are no reports concerning the impact of climatotherapy on long COVID symptoms. Nevertheless, staying in a climate proven to have healing properties may also positively act on the long COVID symptoms.

Physical medicine modalities were applied, excluding balneological factors and exercises, in people undergoing health resort treatment. Low-level laser therapy was administered to 59.02% of participants. This modality is proven to stimulate various effects, including pain reduction, anti-inflammatory effects, regeneration, tissue proliferation, and vasodilatation. However, those effects are claimed to be local [54,55,56]. Local cryotherapy was performed in 50.82% of patients. This modality is claimed to reduce pain, decrease inflammation, diminish oedema, and reduce nerve conduction velocity [57,58]. Electromagnetic fields and electrotherapy (mainly transcutaneous electrical nerve stimulation, TENS) were applied less frequently in the study group. The electromagnetic fields reveal various biological effects, including pain relief, anti-inflammatory, angiogenesis, extensive vasodilatation, tissue regeneration, and proliferation [59], while transcutaneous electrical nerve stimulation is claimed to have a significant analgesic effect [60]. A proportion of 22.13% of participants had been diagnosed with degenerative joint disease. Physical medicine modalities have been proven effective in treating osteoarthritis [55,58,61,62,63]. Even though applied physical medicine modalities act locally, they improve functioning and may have led to improved outcomes in the study group.

## 5. Study Limitations and Further Studies Directions

This study has some limitations. Firstly, the assessment of symptom severity was subjective. Objective methods should be introduced in further studies to measure the condition of the cardiopulmonary system. Implementing the study protocol with spirometry, ECG, a 6 min walk, or a stress test would be reasonable. Secondly, we did not assess the direct interval between SARS-CoV-2 infection and health resort initiation. In further studies, it would be essential to assess this precisely. Thirdly, we did not perform blood sampling. As it is assumed that health resort treatment reduces the level of C-reactive protein, it would be recommended to examine blood morphology, the levels of inflammatory markers, the glucose level, and HbA_1C_ in diabetic patients in further studies. Fourthly, due to age-related comorbidities, e.g., hypertension, it is difficult to clearly establish whether the declared symptoms arose directly from long COVID or comorbidities. In further studies, participants ought to be divided into subgroups depending on the presence of comorbidities and the drugs taken should be considered. From our point of view, further studies might be designed as a clinical trial with long COVID patients divided into groups with different treatment courses to establish the most effective approach. In further studies, the assessment of peripheral resistance might be implemented as well.

## 6. Conclusions

The persistent symptoms of COVID-19 are of low intensity in people qualified for health resort treatment and are independent of gender. Health resort treatment effectively mitigates dyspnoea, tightness of chest, and sputum in long COVID patients. Resort treatment should be implemented into the standard treatment course for COVID-19 survivors as a continuation of therapy.

## Figures and Tables

**Table 1:** Characteristics of the women and men with long COVID undergoing health resort treatment.

	Women (n = 71)			Men (n = 51)		
	Min	Max	Mean ± SD	Min	Max	Mean ± SD
Age [years]	43	77	65.00 ± 8.59	42	79	63.45 ± 8.78
Body weight [kg]	55	125	82.18 ± 16.14	70	120	90.66 ± 12.36
Body height [m]	1.5	1.78	1.62 ± 0.05	1.61	1.95	1.75 ± 0.07
BMI [kg/m^2^]	21.45	46.55	31.32 ± 5.89	23.99	39.79	29.7 ± 3.51
Systolic blood pressure [mmHg]	105	160	139.22 ± 14.36	115	165	140.52 ± 11.7
Diastolic blood pressure [mmHg]	55	95	78.97 ± 8.04	66	95	81.59 ± 6.49
Treatment duration [day]	19	47	25.21 ± 6.71	17	46	23.72 ± 5.74
Comorbidities		Women (n = 71)		Men (n = 51)		
Hypertension [n]		44		26		
Osteoarthritis [n]		16		11		
Type 2 diabetes mellitus [n]		13		10		
Hypothyroidism [n]		14		2		
Gout [n]		2		2		
Benign prostatic hyperplasia [n]		- -		1		

**Table 2:** Comparison of the age-dependent groups of patients undergoing health resort treatment.

	Group I (n = 37)		Group II (n = 42)		Group III (n = 43)		*p* ^1^ Value	Post-Hoc Test ^2^
	Min Max	Mean ± SD	Min Max	Mean ± SD	Min Max	Mean ± SD		
Body weight [kg]	62 125	87.97 ± 14.36	57 120	89.33 ± 17.6	55 110	80.28 ± 11.83	0.02	Group II > Group III *p* = 0.02
Body height [m]	1.51 1.95	1.7 ± 0.09	1.52 1.88	1.68 ± 0.08	1.5 1.78	1.64 ± 0.07	0.04	Group I > Group III *p* = 0.005 Group II vs. Group III: *p* = 0.03
BMI [kg/m^2^]	21.97 46.47	30.49 ± 4.9	21.45 46.65	31.45 ± 5.79	22.03 40.48	29.98 ± 4.47	0.73	-
Systolic blood pressure [mmHg]	105 165	135.32 ± 14.06	109 160	140.16 ± 12.29	115 160	143.2 ± 12.7	0.03	Group I < Group III *p* = 0.02
Diastolic blood pressure [mmHg]	66 95	83.03 ± 6.79	55 95	79.81 ± 7.51	63 95	77.77 ± 7.41	0.02	Group I > Group III *p* = 0.006
Treatment duration [day]	17 41	23.67 ± 5.49	20 46	25.02 ± 6.57	19 47	24.95 ± 6.83	0.38	-

Legend: *p* ^1^—Kruskal–Wallis one-way analysis of variance by ranks; post-hoc test ^2^—the *p* values are presented below the information about differences between the groups.

**Table 3:** Health resort treatment-mediated severity of persistent COVID-19 symptoms.

	Before Resort Treatment			After Resort Treatment			*p* ^1^ Value
	Min	Max	Mean ± SD	Min	Max	Mean ± SD	
Dyspnoea at rest [points]	0	6	1.11 ± 1.51	0	3	0.31 ± 0.62	<0.0001
Exercise-induced dyspnoea [points]	0	9	3.20 ± 2.73	0	4	1.19 ± 1.27	<0.0001
Cough intensity [points]	0	6	0.96 ± 1.41	0	2	0.18 ± 0.46	<0.0001
Tightness of the chest [points]	0	6	0.75 ± 1.47	0	4	0.20 ± 0.6	<0.0001
Chest pain [points]	0	6	0.34 ± 0.96	0	2	0.08 ± 0.3	0.002
Sputum [points]	0	5	0.69 ± 1.14	0	3	0.13 ± 0.44	<0.0001
Palpitations [points]	0	5	0.22 ± 0.76	0	3	0.07 ± 0.37	0.02
Fast heart rate [points]	0	5	0.24 ± 0.81	0	3	0.08 ± 0.38	0.06

Legend: *p* ^1^—difference between measurements.

**Table 4:** The severity of persistent COVID-19 symptoms during the pre- and post-treatment measurements in women and men.

	Dyspnoea at Rest [Points] (Mean ± SD)		
	Before Resort Treatment	After Resort Treatment	*p* ^1^ Value
Women (n = 71)	1.2 ± 1.46	0.39 ± 0.73	<0.0001
Men (n = 51)	0.98 ± 1.58	0.2 ± 0.4	<0.0001
*p* ^2^ value	0.15	0.24	
Exercise-induced dyspnoea [points] (Mean ± SD)			
	Before resort treatment	After resort treatment	*p* ^1^ value
Women (n = 71)	3.13 ± 2.75	1.15 ± 1.21	<0.0001
Men (n = 51)	3.31 ± 2.74	1.23 ± 1.35	<0.0001
*p* ^2^ value	0.69	0.83	
Cough intensity [points] (Mean ± SD)			
	Before resort treatment	After resort treatment	*p* ^1^ value
Women (n = 71)	1.15 ± 1.62	0.21 ± 0.5	<0.0001
Men (n = 51)	0.69 ± 1.01	0.14 ± 0.4	<0.0001
*p* ^2^ value	0.2	0.42	
Tightness of the chest [points] (Mean ± SD)			
	Before resort treatment	After resort treatment	*p* ^1^ value
Women (n = 71)	0.89 ± 1.63	0.22 ± 0.7	<0.0001
Men (n = 51)	0.57 ± 1.19	0.18 ± 0.43	0.04
*p* ^2^ value	0.17	0.72	
Chest pain [points] (Mean ± SD)			
	Before resort treatment	After resort treatment	*p* ^1^ value
Women (n = 71)	0.25 ± 0.73	0.08 ± 0.33	0.04
Men (n = 51)	0.47 ± 1.2	0.08 ± 0.27	0.04
*p* ^2^ value	0.51	0.89	
Sputum [points] (Mean ± SD)			
	Before resort treatment	After resort treatment	*p* ^1^ value
Women (n = 71)	0.75 ± 1.19	0.14 ± 0.49	<0.0001
Men (n = 51)	0.61 ± 1.06	0.12 ± 0.38	<0.0001
*p* ^2^ value	0.53	0.98	
Palpitations [points] (Mean ± SD)			
	Before resort treatment	After resort treatment	*p* ^1^ value
Women (n = 71)	0.28 ± 0.85	0.09 ± 0.45	0.03
Men (n = 51)	0.14 ± 0.63	0.04 ± 0.2	0.62
*p* ^2^ value	0.16	0.65	
Fast heart rate [points] (Mean ± SD)			
	Before resort treatment	After resort treatment	*p* ^1^ value
Women (n = 71)	0.28 ± 0.85	0.14 ± 0.49	0.34
Men (n = 51)	0.18 ± 0.76	0 ± 0	0.13
*p* ^2^ value	0.29	0.02	

Legend: *p* ^1^—difference between measurements; *p* ^2^—difference between sexes.

**Table 5:** The severity of persistent COVID-19 symptoms during the pre- and post-treatment measurements in the age groups.

	Dyspnoea at Rest [Points] (Mean ± SD)		
	Before Resort Treatment	After Resort Treatment	*p* ^1^ Value
Group I (n = 37)	1.49 ± 1.04	0.49 ± 0.84	<0.0001
Group II (n = 42)	1.23 ± 0.98	0.59 ± 0.79	<0.0001
Group III (n = 43)	1.39 ± 0.85	0.49 ± 0.55	<0.0001
*p* ^2^ value	0.54	0.61	
Exercise-induced dyspnea [points] (Mean ± SD)			
	Before resort treatment	After resort treatment	*p* ^1^ value
Group I (n = 37)	3.92 ± 2.95	1.43 ± 1.36	<0.0001
Group II (n = 42)	3.02 ± 2.87	1.05 ± 1.25	<0.0001
Group III (n = 43)	2.77 ± 2.31	1.12 ± 1.19	<0.0001
*p* ^2^ value	0.20	0.37	
Cough intensity [points] (Mean ± SD)			
	Before resort treatment	After resort treatment	*p* ^1^ value
Group I (n = 37)	1.38 ± 1.77	0.24 ± 0.55	<0.0001
Group II (n = 42)	0.59 ± 0.79	0.09 ± 0.3	<0.0001
Group III (n = 43)	0.95 ± 1.46	0.21 ± 0.51	<0.0001
*p* ^2^ value	0.29	0.44	
Tightness of the chest [points] (Mean ± SD)			
	Before resort treatment	After resort treatment	*p* ^1^ value
Group I (n = 37)	1.27 ± 1.83	0.24 ± 0.72	0.002
Group II (n = 42)	0.45 ± 1.33	0.12 ± 0.33	0.44
Group III (n = 43)	0.6 ± 1.11	0.25 ± 0.69	0.003
*p* ^2^ value	0.05	0.83	
Chest pain [points] (Mean ± SD)			
	Before resort treatment	After resort treatment	*p* ^1^ value
Group I (n = 37)	0.57 ± 1.12	0.11 ± 0.31	0.01
Group II (n = 42)	0.21 ± 0.98	0.05 ± 0.21	1.00
Group III (n = 43)	0.28 ± 0.76	0.09 ± 0.37	0.13
*p* ^2^ value	0.1	0.6	
Sputum [points] (Mean ± SD)			
	Before resort treatment	After resort treatment	*p* ^1^ value
Group I (n = 37)	0.7 ± 1.24	0.08 ± 0.36	0.003
Group II (n = 42)	0.45 ± 0.7	0.05 ± 0.21	<0.0001
Group III (n = 43)	0.91 ± 1.34	0.25 ± 0.62	<0.0001
*p* ^2^ value	0.34	0.06	
Heart palpitations [points] (Mean ± SD)			
	Before resort treatment	After resort treatment	*p* ^1^ value
Group I (n = 37)	0.4 ± 0.98	0.11 ± 0.39	0.04
Group II (n = 42)	0.05 ± 0.21	0.07 ± 0.34	1.00
Group III (n = 43)	0.21 ± 0.83	0.09 ± 0.48	0.37
*p* ^2^ value	0.11	0.5	
Fast heart rate [points] (Mean ± SD)			
	Before resort treatment	After resort treatment	*p* ^1^ value
Group I (n = 37)	0.43 ± 1.09	0.05 ± 0.23	0.04
Group II (n = 42)	0.12 ± 0.45	0.02 ± 0.15	0.62
Group III (n = 43)	0.19 ± 0.79	0.16 ± 0.57	0.62
*p* ^2^ value	0.21	0.37	

Legend: *p* ^1^—difference between measurements; *p* ^2^—difference between groups.

**Table 6:** The severity of persistent COVID-19 symptoms during the pre- and post-treatment measurements with respect to the treatment strategy.

	Dyspnoea at Rest [Points] (Mean ± SD)		
	Before Resort Treatment	After Resort Treatment	*p* ^1^ Value
Group A (n = 73)	1.26 ± 1.5	0.31 ± 0.63	<0.0001
Group B (n = 49)	0.88 ± 1.51	0.31 ± 0.62	0.005
*p* ^2^ value	0.25	0.85	
Exercise-induced dyspnoea [points] (Mean ± SD)			
	Before resort treatment	After resort treatment	*p* ^1^ value
Group A (n = 73)	2.85 ± 2.71	1.03 ± 1.28	<0.0001
Group B (n = 49)	3.74 ± 2.71	1.43 ± 1.22	<0.0001
*p* ^2^ value	0.12	0.04	
Cough intensity [points] (Mean ± SD)			
	Before resort treatment	After resort treatment	*p* ^1^ value
Group A (n = 73)	1.00 ± 1.35	0.16 ± 0.44	<0.0001
Group B (n = 49)	0.89 ± 1.5	0.2 ± 0.49	<0.0001
*p* ^2^ value	0.33	0.68	
Tightness of the chest [points] (Mean ± SD)			
	Before resort treatment	After resort treatment	*p* ^1^ value
Group A (n = 73)	0.78 ± 1.54	0.23 ± 0.67	<0.0001
Group B (n = 49)	0.71 ± 1.37	0.16 ± 0.47	0.02
*p* ^2^ value	0.93	0.66	
Chest pain [points] (Mean ± SD)			
	Before resort treatment	After resort treatment	*p* ^1^ value
Group A (n = 73)	0.26 ± 0.82	0.11 ± 0.35	0.18
Group B (n = 49)	0.47 ± 1.14	0.04 ± 0.19	0.008
*p* ^2^ value	0.22	0.25	
Sputum [points] (Mean ± SD)			
	Before resort treatment	After resort treatment	*p* ^1^ value
Group A (n = 73)	0.76 ± 1.18	0.11 ± 0.46	<0.0001
Group B (n = 49)	0.57 ± 1.06	0.16 ± 0.42	0.02
*p* ^2^ value	0.22	0.19	
Palpitations [points] (Mean ± SD)			
	Before resort treatment	After resort treatment	*p* ^1^ value
Group A (n = 73)	0.25 ± 0.86	0.09 ± 0.45	0.04
Group B (n = 49)	0.18 ± 0.60	0.04 ± 0.19	0.37
*p* ^2^ value	0.88	0.71	
Fast heart rate [points] (Mean ± SD)			
	Before resort treatment	After resort treatment	*p* ^1^ value
Group A (n = 73)	0.23 ± 0.91	0.09 ± 0.41	0.28
Group B (n = 49)	0.24 ± 0.66	0.06 ± 0.31	0.22
*p* ^2^ value	0.44	0.55	

Legend: *p* ^1^—difference between measurements; *p* ^2^—difference between groups.

## Data Availability

The data that support the findings of this study are available from the corresponding author.
